# Working memory, age and education: A lifespan fMRI study

**DOI:** 10.1371/journal.pone.0194878

**Published:** 2018-03-27

**Authors:** Jo A. Archer, Annie Lee, Anqi Qiu, S-H Annabel Chen

**Affiliations:** 1 Psychology, Nanyang Technological University, Singapore, Singapore; 2 Department of Biomedical Engineering, National University of Singapore, Singapore, Singapore; 3 Clinical Imaging Research Centre, National University of Singapore, Singapore, Singapore; 4 Singapore Institute for Clinical Sciences, the Agency for Science, Technology and Research, Singapore, Singapore; 5 Centre for Research and Development in Learning, Nanyang Technological University, Singapore, Singapore; 6 Lee Kong Chian School of Medicine, Nanyang Technological University, Singapore, Singapore; Chinese Academy of Sciences, CHINA

## Abstract

Ageing is associated with grey matter atrophy and changes in task-related neural activations. This study investigated the effects of age and education on neural activation during a spatial working memory task in 189 participants aged between 20–80 years old, whilst controlling for grey matter density. Age was related to linear decreases in neural activation in task activated areas, and this effect was no longer significant when adjusting for education or accuracy. Age was also related to cubic increases in neural activation in non-task related areas, such as the temporal gyrus, cuneus and cerebellum when adjusting for accuracy and education. These findings support previous lifespan datasets indicating linear age-related decreases in task activation, but non-linear increases in non-task related areas during episodic memory tasks. The findings also support past studies indicating education offers a form of cognitive reserve through providing a form of neural compensation and highlights the need to consider education in ageing studies.

## Introduction

Ageing has been associated with cognitive decline and anatomical and functional neural changes [1–4]. Variance in task performance, neural volume and activity also increases with age [3].

The majority of studies investigating the neural effects of ageing have compared older adults to younger adults (see [5, 6]). However, some researchers have investigated lifespan changes in brain volume [7, 8] and task activity [9–12]. The studies on changes in brain volume indicate the association between grey matter volume and age varies depending on the area of the brain [7, 8]. One of the first functional imaging studies to include a middle age group demonstrated that age was linearly associated with less deactivation in medial areas, such as the precuneus, medial frontal gyri and cingulate gyri (i.e. activity in these areas increased with age, but these areas are normally deactivated in the task) [9]. They also found a linear decrease in occipital, caudal and right middle frontal activity, but task performance was not accounted for and only linear models were tested. However, a more recent study also showed increased activation in medial areas, implicated in the default mode network, with age, despite no association between age and accuracy [10]. Jamadar and colleagues (2013) demonstrated increased activation with age during recognition, but only some of these effects remained significant after controlling for grey matter volume, supportive of a combination of neural reserve and neural compensation [11]. These authors also showed non-linear associations (mainly quadratic) between age and neural activation in non-task related areas with no distinctive pattern to the areas showing non-linear associations. Finally, Trivedi and colleagues (2008) observed linear age-related decreases in neural activation in task areas in a large sample during episodic memory encoding and self-appraisal tasks [12]. These studies have made great contributions towards the understanding of age-related changes in task performance. However, only one of these studies [11] adjusted for grey matter volume and the adjustment was carried out as whole brain grey matter volume, which given the regional differences in age-related grey matter volume loss [7, 8] may result in over adjustment in some areas, but under adjustment in others. In addition, only Trivedi and colleagues (2008) [12] adjusted for education despite evidence showing education is often lower in older compared to younger adults and is associated with better cognitive performance [13]. No studies accounted for both grey matter volume and education.

The previous lifespan studies mentioned above employed visual and verbal episodic memory encoding tasks. Cognitive data has shown that visuospatial working memory ability also declines with age, often proving to be as, if not more, sensitive than language dominant tasks [14, 15]. Our current study therefore applied a visuospatial working memory task as a comparison to previous studies investigating episodic memory. The aims of this study are two-fold: (1) to identify age-related changes in neural function across the lifespan whilst sensitively adjusting for grey matter volume and (2) to investigate age-related changes in neural function whilst adjusting for education. It is hypothesised that (1) increasing age will be associated with decreased education and task accuracy; (2) increasing age will be associated with decreased activation of task-related areas; (3) the association between age and decreased neural function will no longer be significant after adjusting for education or accuracy.

## Methods

### Participants

Two-hundred and ten participants aged 21 to 79 years old who reported none of the following conditions were recruited: (1) major illnesses/surgery (heart, brain, kidney, lung surgery); (2) neurological or psychiatric disorders; (3) learning disability or attention deficit; (4) previous head injury with loss of consciousness; (5) non-removable metal objects on/in the body such as cardiac pacemaker; (6) diabetes or obesity; (7) a Mini-Mental State Examination (MMSE) score of less than 24 [16]. Information on highest education level was self-reported as none, primary school, secondary school, secondary school qualification, higher education qualification, degree level qualification and above. Only nine participants reported lower than secondary school education, so education was recoded as: up to secondary school; secondary school qualification; higher education qualification; degree level qualification and above.

The study was granted ethical approval according to the Declaration of Helsinki by the National University of Singapore Institutional Review Board. All participants were informed and gave written consent prior to the start of the study.

### Neuropsychological tests

#### The Repeatable Battery for the Assessment of Neuropsychological Status (RBANS)

The Repeatable Battery for the Assessment of Neuropsychological Status (RBANS) is a cognitive screening battery which has shown high validity in identifying cognitive impairment, decline or improvement in community populations [17, 18]. The RBANS consists of 12 tests which are split into five domains. The raw scores for the five domains were used in this study, representing: immediate memory (IM), visuospatial/construction (VC), delayed memory (DM), language (Lang), attention (Att) and delayed memory (DM).

#### The Cambridge Neuropsychological Test Automated Battery (CANTAB)

The Cambridge Neuropsychological Test Automated Battery (CANTAB) has been extensively validated for assessing cognitive performance in healthy ageing samples and neuropsychiatric populations [19, 20]. The relationship between age, education and CANTAB performance for this sample has been explored in greater depth in a previous study [21]. The visuospatial paired associate learning (PAL) task was selected for inclusion in this study as a comparison to the fMRI task performance because it has previously been shown to be sensitive to age-related cognitive decline [19, 21]. Moreover, the PAL task has been shown to be more sensitive to age-related cognitive decline compared to other CANTAB tasks in both the present sample and other populations [21].

The task involves correctly associating a shape with a location. Six white boxes are displayed on the computer screen. During a trial the boxes are ‘opened’ in a randomised order to reveal a pattern. Participants are then presented with the patterns in the middle of the screen and are required to select the location where the pattern previously appeared. No time limit is enforced and if a participant answers correctly the number of patterns presented increases; starting at one, then two, three, then six and finishing at eight patterns. An error results in a repeat of the same trial without increasing the pattern number. The task stops when a participant fails to recall the correct locations after 10 attempts. The first trial memory score is calculated as the number of patterns correctly located in the first attempt of that trial, resulting in a maximum score of 26 with higher scores indicating better associative memory.

### fMRI experimental design and procedure

**Fig 1:**
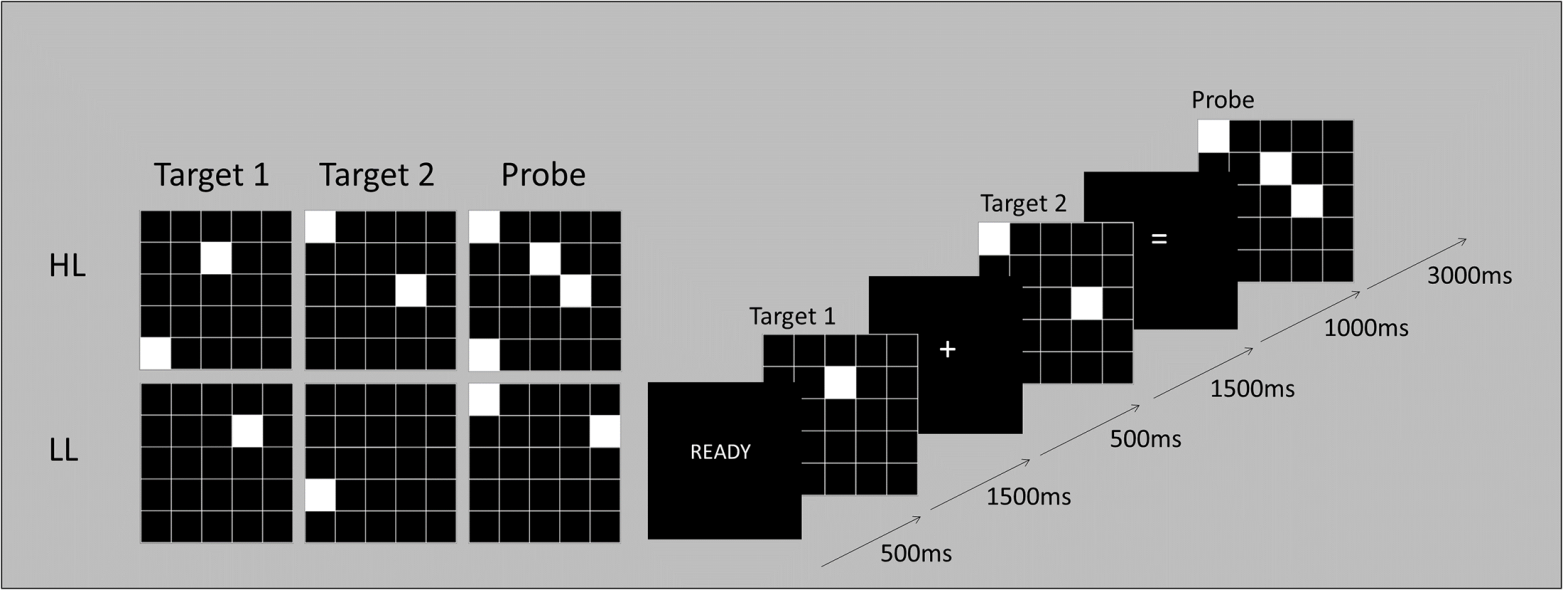
The spatial addition task. HL = high load, LL = low load. Arrows indicate length of presentation. Participants determined if the shaded squares in the Probe were in the same positions as Target 1 and Target 2 combined, responding with a button press during the Probe period.

The Spatial Addition Task (SAT) is a visuospatial working memory task that allows evaluation of processes involved in maintenance only and maintenance plus processing. There were four conditions in this task: Low Load (LL); High Load (HL); Low Maintenance (LM); High Maintenance (HM) (Fig 1). The contrast HM>LM represented maintenance load and the contrast HL>LL represented manipulation load. However, based on accuracy means, whilst performance for the HL and LL condition was consistent with expectations, participants found the HM condition easier than the LM condition which was contrary to expectations and posthoc tests indicated a significant difference (*p* <0.001). The conditions were modelled separately at first-level analyses, and as a precaution, the HM>LM contrast was not included in any subsequent analyses.

In each condition, the word “ready” was presented for 500ms to cue participants to the start of a trial. This was followed by the first target—a 5x5 square matrix formed of white lines on a black background where either one or two of the squares was shaded in white—which was shown for 1500ms, followed by an interstimulus interval (fixation cross) of 500ms. Participants were then shown another 5x5 matrix with one or two shaded squares (Target 2). An “equals” sign was then displayed for 1000ms, representing the maintenance period, after which another 5x5 matrix was shown for 3000ms (the Probe). For half of the trials in each condition the probe correctly represented the addition of Target 1 and Target 2 (correct) and in the other half the probe did not represent the addition of Target 1 and Target 2 (incorrect). Subjects determined if the shaded squares in the Probe were in the same positions as Target 1 and Target 2 combined, responding with a button press during the Probe period using left thumb if the Probe was the spatial sum of Target 1 and Target 2 and their right thumb if the Probe was not the sum of the previous Targets. Only one square was shaded in each Target for LL, whereas two squares were shaded for the HL condition. The presentation of correct and incorrect trials was randomised. Each trial lasted for eight seconds, and each block included two trials. Each run included three cycles lasting a total of 192s (3 mins 12s). Participants completed a practice task which included feedback before entering the scanner and completed three runs in the scanner without feedback.

The task activated visuospatial and working memory networks [22] and performance was generally good (see Results and Table 1).

**Table 1:** Task performance in the final sample.

	Condition		Significance
	HL	LL	
Accuracy	0.92 (0.08)	0.94 (0.08)	t(188) = -4.05, *p* < .001
RT	1020.75 (247.78)	862.54 (202.20)	t(188) = 16.23, *p* < .001

HL = high load, LL = low load, RT = reaction time

### Functional imaging acquisition

Images were acquired with a Siemens 3T Trio MRI scanner using a 32-channel quadrature headcoil at the Clinical Imaging Research Centre, National University of Singapore. Whole brain structural scans were obtained prior to functional Magnetic Resonance Imaging (fMRI), consisting of an MP-RAGE anatomical sequence (192 axial slices of 1mm thickness, repetition time (TR) = 2300ms, echo time (TE) = 1.9ms, flip angle (FA) = 9°, field of view (FOV) = 256mm, matrix = 256x256, interleaved acquisition). Whole brain fMRI data were obtained using an Echo Planar Imaging (EPI) sequence (48 axial slices of 3mm thickness with no gap, TR = 2400ms, TE = 25ms, FA = 90°, FOV = 192mm, matrix = 64x64, interleaved acquisition). All images were acquired co-planar with the anterior commissure—posterior commissure line and the first three images of any run were discarded. A total of 80 images were acquired for each SAT run. Runs with lower than 60% accuracy in any condition were excluded from the analyses, as were runs which included greater than 1.5mm translational movement or more than 2° rotation.

### Procedure

First, participants completed a session of questionnaires and neuropsychological tests. This was followed by MRI scanning, starting with structural scans, then a resting-state scan and task scans. All participants completed three runs of the SAT task. Stimuli were presented using Eprime v2.0 [23] and accuracy and response times (RTs) were recorded.

### Statistical analysis

#### Behavioural data

A Bonferroni corrected correlation matrix was conducted to investigate the relationship between age, education, neuropsychological task performance and SAT accuracy. A second correlation matrix was conducted to investigate the relationship between age, neuropsychological task performance and SAT accuracy adjusting for education, using linear regression and a Bonferroni adjusted *p* value.

T-tests were conducted to test for differences in accuracy and RTs between the task conditions. Regression analyses were conducted to investigate the relationship between age and task performance (accuracy and RTs for each condition). Condition was entered as an interaction term to investigate if condition moderated the relationship between age and task performance. Likelihood Ratio tests were conducted to investigate if quadratic and cubic associations between age and task performance contributed to the model. Regression analyses were also conducted to investigate the relationship between education and task performance, and condition was entered as an interaction term to investigate if condition moderated the relationship between education and task performance. All behavioural analyses were conducted in Stata v11.2 [24].

#### fMRI data

**Preprocessing:** Images were preprocessed in Statistical Parametric Mapping 8 (SPM8, Wellcome Department of Imaging Neuroscience, London, UK http://www.fil.ion.ucl.ac.uk/spm) in MATLAB v7.9.0 [25] using the steps (1) slice timing correction (to the middle slice using Fourier phase shift interpolation), (2) realignment for motion correction (to first image), (3) coregistration (using entropy correlation coefficient) and (4) the Diffeomorphic Anatomical Registration Through Exponentiated Lie algebra (DARTEL) pipeline [26] was applied to obtain a group specific structural template for segmentation and normalisation to the standard (Montreal Neurological Institute 152, MNI) space and smoothing using an 8x8x8mm full width half maximum (FWHM) Gaussian kernel. The grey matter probability (GMP) maps obtained in segmentation were resliced to a voxel dimension of 3x3x3, normalised and Jacobian modulated and smoothed using an 8x8x8mm FWHM Gaussian Kernel so as to match the functional data to provide a grey matter density map which was used as a covariate in the second level analyses.

**First level analysis:** A fixed effect general linear model at single subject level was conducted in SPM8 to obtain the task activation contrasts of interest. The SAT task design was a block design with a regressor for each condition. All correct and incorrect trials were included within the same regressor. The task design function was convolved with a canonical haemodynamic response function as the main effect of interest and motion parameters were included as covariates. Low frequency variation was eliminated using a 128-second high pass filter and a one-lag autoregression model was applied globally.

**Second level analysis:** First, one-sample t-tests were conducted in SPM8 to obtain areas activated and deactivated during the task. Second, the effect of age on task activations was investigated in Robust Biological Parametric Mapping (BPM) [27] using robust (Huber) random effect analyses. Linear, quadratic and cubic associations were tested. Grey matter probability was included as a covariate. Third, the effect of age on task activations was conducted employing task activations and deactivations as a mask and GMP as a covariate. Fourth, the effect of age on task activations was conducted with education and GMP as a covariate. Fifth, the effect of age on task activations was conducted with education and the sum of HL and LL accuracy and GMP as a covariate. The voxel threshold of *p* < .001 and cluster threshold of *p* < .05 family wise error corrected (*p* < .05_FWE_) was employed for all second-level imaging analyses.

Anatomical labels were identified by converting MNI coordinates to Talairach using a non-linear transform [28] and referencing the coordinates in the Talairach Atlas [29]. Cerebellum activations were located using MNI coordinates and the Schmahmann and colleagues [30] cerebellar atlas. All coordinates are reported in MNI space.

## Results

### Participants

**Fig 2:**
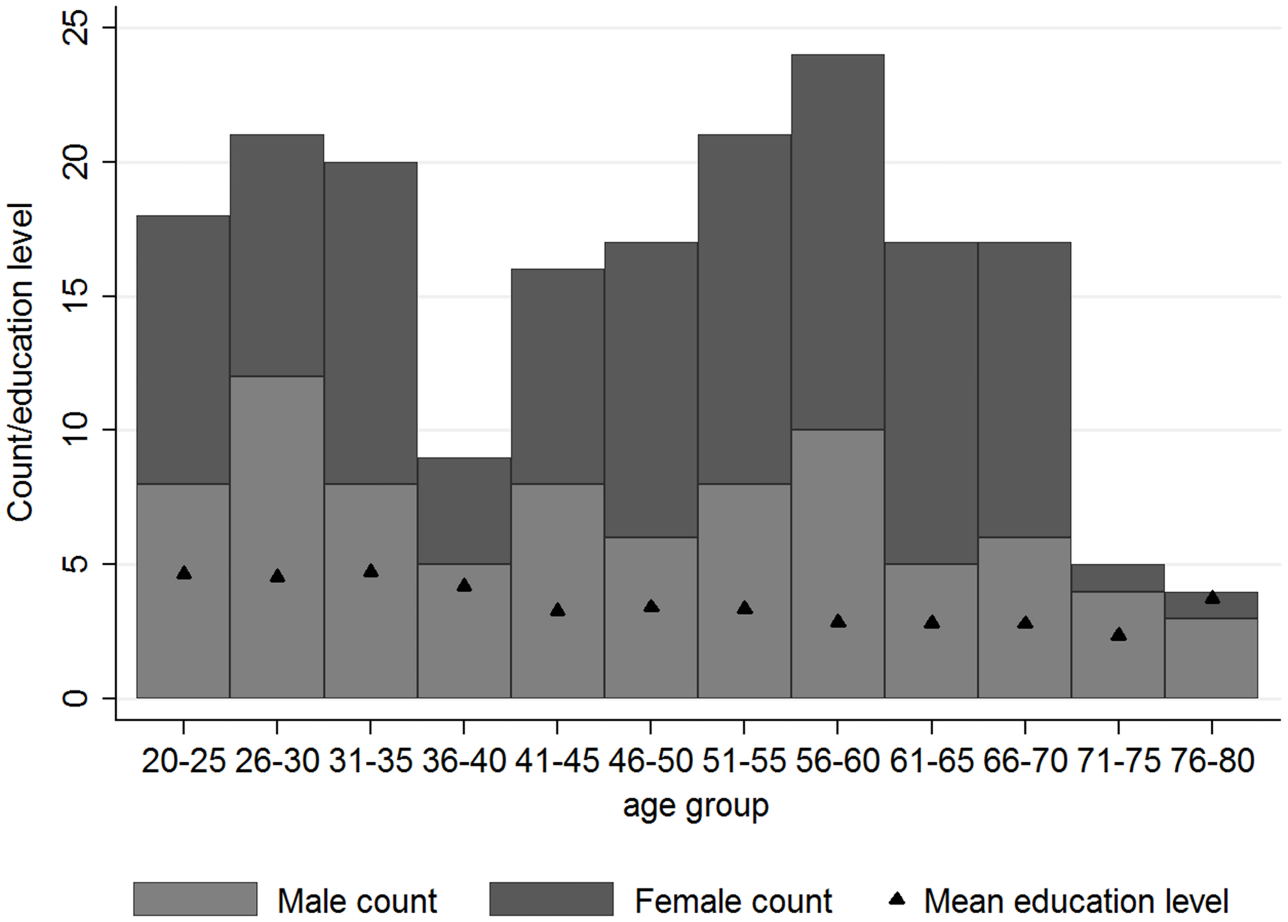
The sample.

Ten participants did not have complete imaging or accuracy data due to technical faults or the participant declined to complete all components of the study. Eleven participants were excluded completely due to either extensive movement or below 60% accuracy in all three runs (age range 42–74 years old), thus the final sample consisted of 189 adults (106 women) aged from 22 to 79 years old (Fig 2). An additional 30 runs from the remaining participants were excluded due to high movement and 34 runs were excluded due to low accuracy. Of the final sample, 163 participants were right handed, 10 left handed and the remainder ambidextrous according to the Edinburgh Handedness Scale [31].

### Task performance

**Fig 3:**
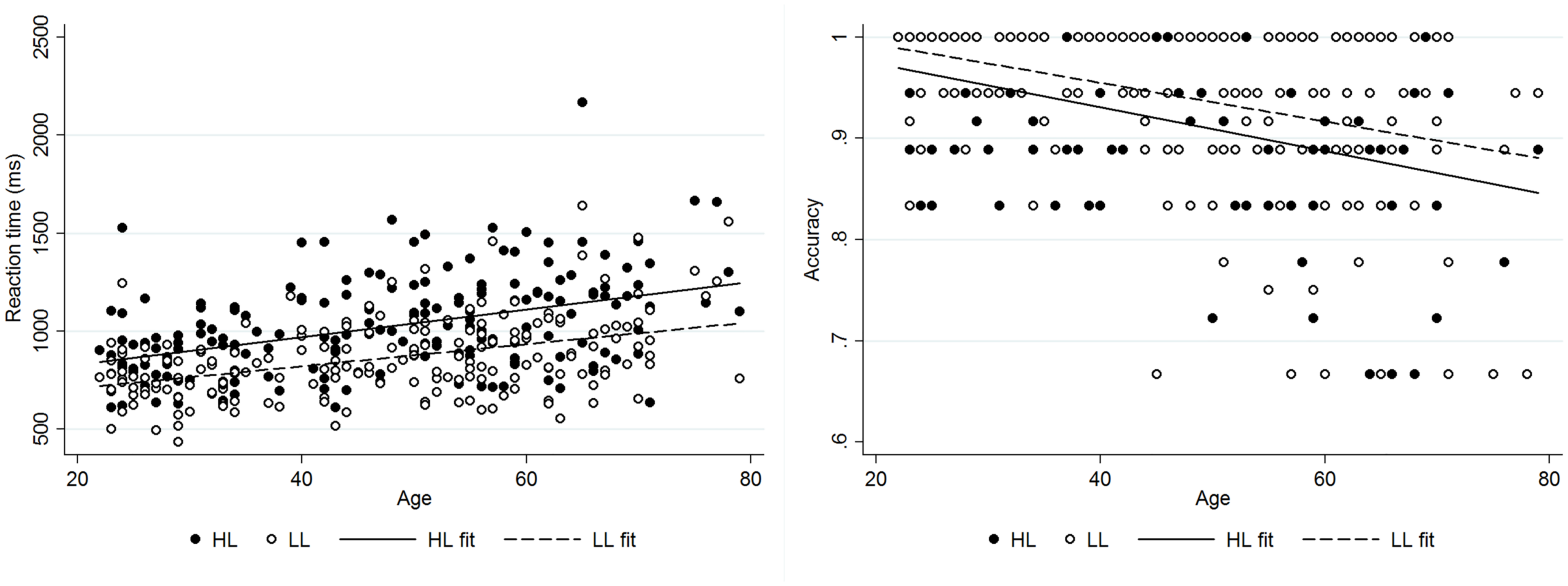
Task performance. HL = high load, LL = low load.

As expected the task activated the visuospatial working memory network [22] (Fig 3). Participants were significantly more accurate and faster in the LL condition compared to the HL condition (Table 1).

### Task performance, education and age

There were significant linear associations between age and task performance; older age was associated with decreased accuracy [β(95% confidence interval, CI) = -.002(-.003, -.001), *p* < .001; β(CI) = -.002(-.003,-.001), *p* < .001 for HL and LL respectively]. Task condition did not significantly moderate the relationship between age and accuracy [β(CI) = .0003(-.001,.001), *p* = .607], as a result, the accuracy for both conditions was summed for future analyses, resulting in a similar association between increasing age and decreasing accuracy [β(CI) = -.004(-.005, -.003), *p* < .001]. Likelihood ratio tests indicated that neither a quadratic or cubic model significantly added anything to the linear model [χ^2^(1) = 3.55, *p* = .060; χ^2^(1) = .57, *p* = .452 respectively]. Older age was also associated with slower response time [β(CI) = 7.06(4.98,9.14), *p* < .001; β(CI) = 5.62(3.91,7.32), *p* < .001 for HL and LL respectively]. Again, task condition did not significantly moderate this relationship [β(CI) = -1.44(-4.12,1.24), *p* = .291], as a result the RT for both conditions was summed for future analyses, resulting in a similar association between increasing age and increasing RT [β(CI) = 12.69(9.08,16.27), *p* < .001]. Likelihood ratio tests indicated that neither a quadratic or cubic model significantly added to the linear model [χ^2^(1) = .34, *p* = .562; χ^2^(1) = .55, *p* = .459 respectively].

Increased education was also associated with a linear increase in accuracy and decrease in RT [β(CI) = .05(.03,.06), *p* < .001; β(CI) = -187.34(-238.93,-135.75), *p* < .001 respectively]. Likelihood ratio tests indicated that quadratic and cubic models did not contribute to the linear model for education and either accuracy or RT [accuracy: χ^2^(1) = 2.55, *p* = .110; χ^2^(1) = .67, *p* = .413; RT: χ^2^(1) = .02, *p* = .879, χ2(1) = .57, *p* = .451 for quadratic and cubic respectively].

### Neuropsychological and fMRI task performance correlations

Scores for the neuropsychological tasks are presented in S1 Fig. The correlations between age, education, SAT performance and neuropsychological test scores are presented in Table 2 and S1 Table. Age was significantly inversely correlated with education, SAT performance and all of the neuropsychological measures. Education was significantly positively correlated with SAT performance and all of the neuropsychological measures except the language domain of the RBANS. SAT performance was significantly positively correlated with all of the neuropsychological measures except the language domain of the RBANS.

**Table 2:** Correlation matrix between age, education, neuropsychological measures and SAT performance.

	age	education	PAL accuracy	IM	VS	Lang	Att	DM
age	**1.000**							
education	**-0.520**	**1.000**						
PAL accuracy	**-0.582**	**0.298**	**1.000**					
IM	**-0.463**	**0.333**	**0.520**	**1.000**				
VS	**-0.251**	**0.343**	**0.246**	**0.260**	**1.000**			
Lang	**-0.249**	0.227	0.231	**0.361**	0.118	**1.000**		
Att	**-0.610**	**0.476**	**0.580**	**0.486**	**0.253**	**0.465**	**1.000**	
DM	**-0.406**	**0.274**	**0.579**	**0.605**	**0.296**	**0.323**	**0.453**	**1.000**
SAT accuracy	**-0.462**	**0.392**	**0.478**	**0.426**	**0.360**	0.230	**0.547**	**0.355**

Standardised betas are reported. Bold text indicates significant to *p*<0.05 Bonferroni corrected. N = 186–189 as some participants did not have valid data for all neuropsychological measures. PAL = paired associate learning, IM = RBANS immediate memory, VS = RBANS visuospatial/construction, Lang = RBANS language, Att = RBANS attention, DM = RBANS delayed memory.

The correlations between age, SAT performance and neuropsychological test scores after adjustment for education are presented in Table 3 and S2 Table. Age remained significantly inversely correlated with SAT performance and the majority of the neuropsychological measures, except for the visuospatial and language domains of the RBANS. Similarly, SAT performance remained significantly positively associated with all of the neuropsychological measures except the language domain of the RBANS.

**Table 3:** Correlation matrix between age, neuropsychological measures and SAT performance adjusting for education.

	age	PAL accuracy	IM	VS	Lang	Att	DM
age	**1.000**						
PAL accuracy	**-0.469**	**1.000**					
IM	**-0.326**	**0.473**	**1.000**				
VS	-0.824	0.163	0.165	**1.000**			
Lang	-0.138	0.173	**0.301**	0.042	**1.000**		
Att	**-0.468**	**0.566**	**0.424**	0.116	**0.462**	**1.000**	
DM	**-0.285**	**0.538**	**0.555**	0.219	**0.282**	**0.348**	**1.000**
SAT accuracy	**-0.395**	**0.427**	**0.349**	**0.266**	0.166	**0.425**	**0.292**

Standardised betas are reported. Bold text indicates significant to *p*<0.05 Bonferroni corrected p value of *p* = 0.0018. N = 186–189 as some participants did not have valid data for all neuropsychological measures. PAL = paired associate learning, IM = RBANS immediate memory, VS = RBANS visuospatial/construction, Lang = RBANS language, Att = RBANS attention, DM = RBANS delayed memory.

### Task activations

**Fig 4:**
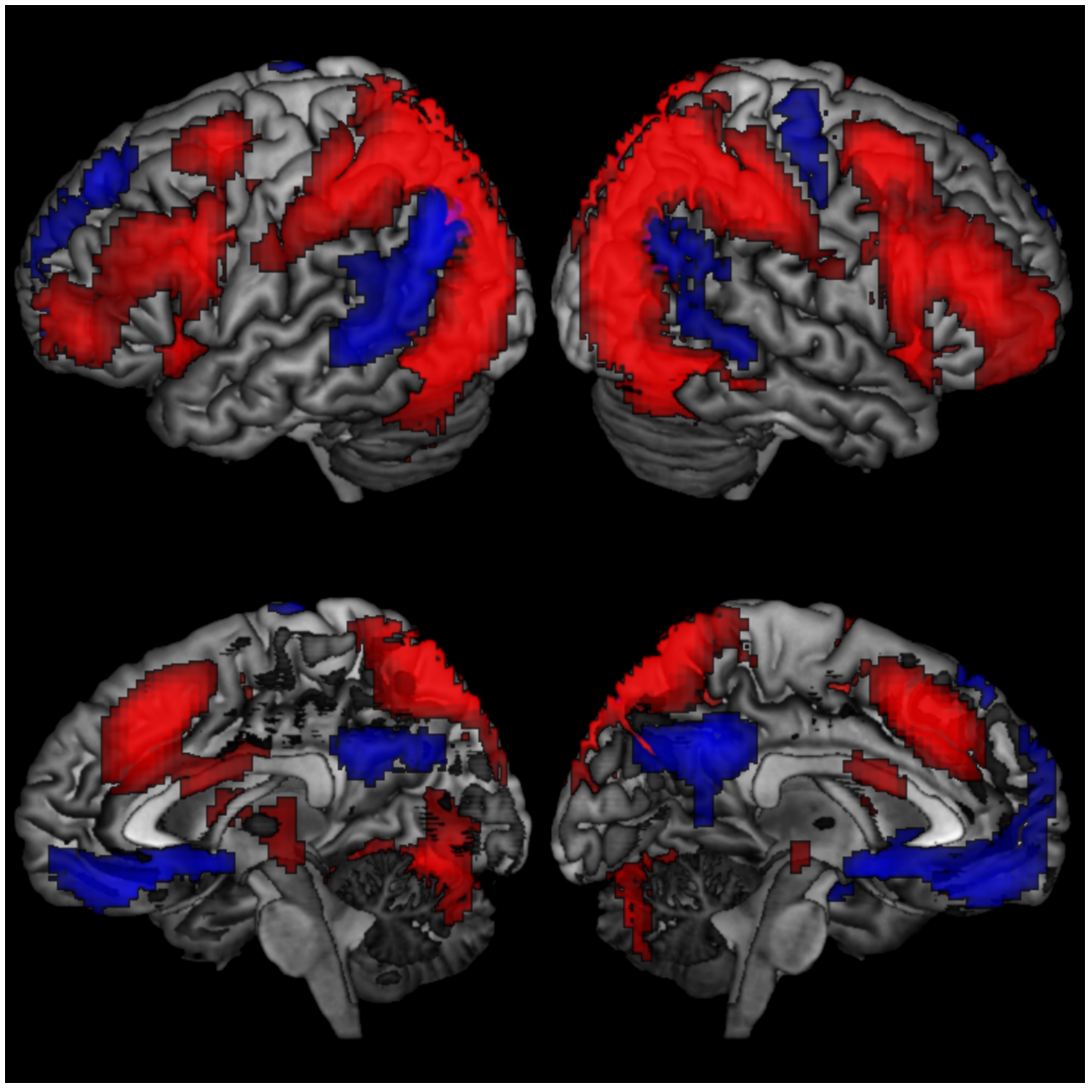
Task activations and deactivations for the whole sample. Red = activation, blue = deactivation. Voxel p < .001, cluster p < .05_(FWE-corrected)_.

A one sample t-test indicated activations in the right precuneus and middle frontal gyrus for the HL>LL contrast, with deactivations in the angular gyri and posterior and anterior cingulate (Table 4, Fig 4, S3 Table).

**Table 4:** Task activations.

	hemi	BA	Cluster size	Peak T	Peak Z	MNI coordinates			cluster P_(FWE)_
						x	y	z	
*Activations*									
Precuneus	R	7	8501	16.29	65535.00	27	-60	45	<0.001
Middle Frontal Gyrus	R	6	6649	11.45	65535.00	27	3	51	<0.001
*Deactivations*									
Angular Gyrus	L	39	721	7.66	7.13	-45	-75	42	<0.001
Cingulate Gyrus	L	31	456	7.48	6.99	-9	-51	33	<0.001
Anterior Cingulate	R	32	1065	6.86	6.47	6	33	-18	<0.001
Angular Gyrus	R	39	154	5.45	5.25	57	-63	33	0.006
Precentral Gyrus	R	4	100	5.10	4.93	39	-18	69	0.032

BA = Brodmann area, hemi = hemisphere, L = left, R = right

### Task activations and age

**Fig 5:**
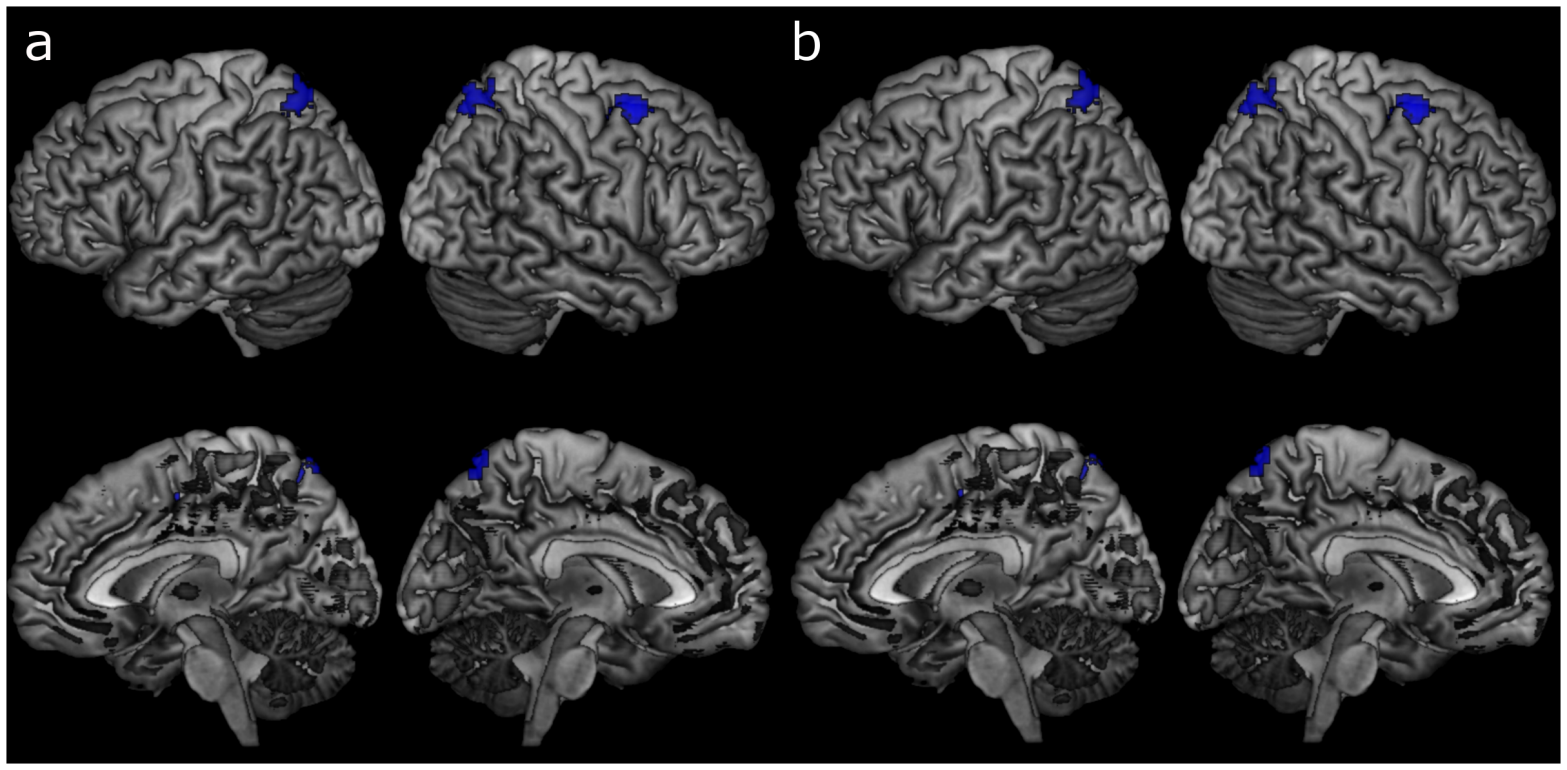
Linear associations between age and task activations adjusted for grey matter probability. **a** unmasked; **b** masked. Blue = decrease in activation with increasing age. Voxel p < .001, cluster p < .05_(FWE-corrected)_.

Age was associated with a linear decrease in activations in the right middle frontal gyrus and bilateral precunei when adjusting for GMP (Table 5, Fig 5a, S4 Table). A similar result was obtained when masked with task activation (Table 5, Fig 5b, S4 Table). Age was not associated with any linear increases in activation. In addition, the significant linear decreases in task activation with increasing age were no longer significant after adjusting for either education or task accuracy. There were no quadratic associations between age and task activations.

**Table 5:** Linear associations between task activations and age.

	hemi	BA	Cluster size	Peak T	Peak Z	MNI coordinates			cluster P_(FWE)_
						x	y	z	
*Unmasked*									
*Adjusting for GMP*									
*Decreased activation with age*									
Middle Frontal Gyrus	R	6	139	5.05	4.88	30	6	60	<0.001
Precuneus	R	7	53	3.91	3.82	21	-69	60	0.009
Precuneus	L	7	49	3.79	3.71	-12	-66	66	0.014
*Masked with task activations*									
*Adjusting for GMP*									
*Decreased activation with age*									
Middle Frontal Gyrus	R	6	137	5.05	4.88	30	6	60	<0.001
Precuneus	R	7	52	3.91	3.82	21	-69	60	0.010
Precuneus	L	7	46	3.79	3.71	-12	-66	66	0.018

BA = Brodmann area, hemi = hemisphere, L = left, R = right

**Fig 6:**
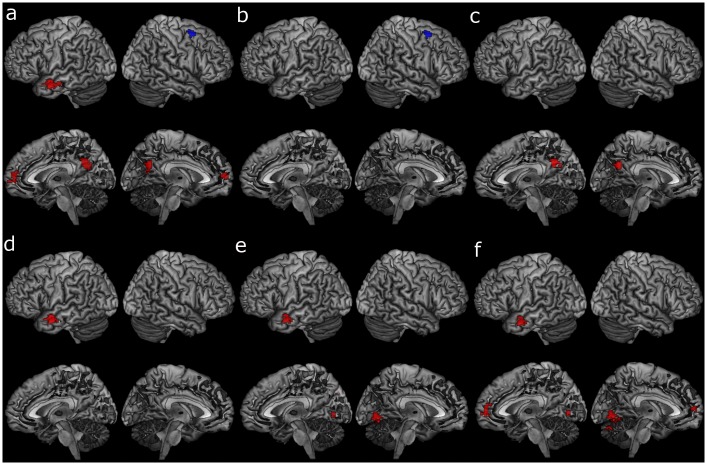
Cubic associations between age and task activations. **a** Cubic association with age, adjusted for GMP; **b** cubic association with age, adjusted for GMP with task positive mask; **c** cubic association with age adjusted for GMP with task negative mask; **d** cubic association with age adjusted for education and GMP; **e** cubic association with age adjusted for education, accuracy and GMP; **f** cubic association with age adjusted for a cubic education level, accuracy and GMP. GMP = grey matter probability, red = increased activation with increasing age, blue = decrease in activation with increasing age. Voxel p < .001, cluster p < .05_(FWE-corrected)_.

Age was also associated with a cubic decrease in activations in the middle frontal gyrus when adjusting for GMP (Table 6, Fig 6a, S5 Table), in a similar area to the linear association, which remained the same when masked with group task activations (Table 4, Fig 6b, S5 Table). In addition, age was associated with a cubic increase in activations in the right precuneus, left middle temporal gyrus and medial frontal gyrus when adjusting for GMP (Table 6, Fig 6a, S5 Table). The association between age and a cubic increase in activations in the right precuneus remained significant when masked with group task deactivations (Table 6, Fig 6c, S5 Table).

**Table 6:** Cubic associations between task activations and age.

	hemi	BA	Cluster size	Peak T	Peak Z	MNI coordinates			cluster P_(FWE)_
						x	y	z	
Unmasked									
*Adjusting for GMP*									
*Increased activation with age*									
Precuneus	R	31	74	5.00	4.84	18	-48	30	0.001
Middle Temporal Gyrus	L	21	107	4.46	4.34	-63	3	-15	0.000
Medial Frontal Gyrus	L	9	60	4.08	3.99	-6	60	9	0.005
*Decreased activation with age*									
Sub-Gyral	R	6	73	4.39	4.27	30	6	57	0.001
*Adjusting for education and GMP*									
*Increased activation with age*									
Superior Temporal Gyrus	L	22	69	4.31	4.20	-51	0	-12	0.002
*Adjusting for education*, *accuracy and GMP*									
*Increased activation with age*									
Cuneus	R	18	58	4.62	4.49	18	-78	21	0.006
Cerebellum, Lobule VI	L		40	4.52	4.39	-9	-66	-6	0.033
Superior Temporal Gyrus	L	38	42	3.90	3.82	-54	0	-12	0.027
*Adjusting for education cubic*, *accuracy and GMP*									
*Increased activation with age*									
Cuneus	R	18	49	4.64	4.50	18	-78	21	0.013
Cerebellum, Lobule VI	L		51	4.53	4.41	-9	-66	-6	0.011
Cerebellum, Lobule VI	L		37	4.29	4.19	-12	-75	-24	0.046
Middle Temporal Gyrus	L	21	45	3.99	3.90	-63	-3	-18	0.020
Medial Frontal Gyrus	L	9	46	3.65	3.58	-6	60	9	0.018
*Masked with task deactivations*									
*Adjusting for GMP*									
*Increased activation with age*									
Precuneus	R	31	48	4.20	4.10	12	-51	36	0.015
*Masked with task activations*									
*Adjusting for GMP*									
*Decreased activation with age*									
Middle Frontal Gyrus	R	6	73	4.39	4.27	30	6	57	0.001

BA = Brodmann area, hemi = hemisphere, L = left, R = right

Age was associated with a cubic increase in activations in the left superior temporal gyrus when adjusting for education and GMP (Table 6, Fig 6d, S5 Table). Age was associated with a cubic increase in activations in the right cuneus, left cerebellum and superior temporal gyrus after adjusting for education, accuracy and GMP (Table 6, Fig 6e, S5 Table). These results remained similar if a cubic transform of education was entered into the model instead of the linear education fitting (Table 6, Fig 6f, S5 Table). None of these associations were significant when masked with either task activations or deactivations.

## Discussion

This study demonstrated linear associations between age, education and task accuracy over the lifespan.

### Behavioural findings

As with many past studies there was an age-related decline in the raw RBANS scores and PAL task performance [17, 19, 21]. Task performance on the SAT was negatively associated with age and positively associated with the neuropsychological scores, with and without adjustment for education. The association between age and poorer task performance also remained for the majority of neuropsychological tasks when adjusting for education. These findings provide support for the validity of the SAT task as an indicator of age-related cognitive decline.

### Ageing

This study replicated previous lifespan neuroimaging studies showing a linear decrease in task activation with increasing age [9–12]. The quadratic associations reported by Jamadar and colleagues (2013) were not replicated, but there were significant cubic increases in neural activations in areas deactivated during the task and outside of the task network [11]. The increases within task-deactivated areas were no longer significant after adjusting for education or accuracy. Nonetheless, some of areas that showed increases in neural activation with age outside of the task network remained significant. Interestingly the age-related decreases in neural activation were linear, within the task network and mediated by education. Notably, the linear decrease in activation with age was not significant after adjusting for accuracy, in keeping with past studies demonstrating that increasing age is associated with linear decreases in cognitive performance from early adulthood [32] and that decreases in activation are related to lower accuracy [33]. In contrast, all the age-related increases in neural activation were non-linear, either within the task negative network or non-task related areas and either partially or completely unaffected by adjustment for education.

Our current study extends previous lifespan study findings [11] by demonstrating that non-linear increases occur in the cuneus, temporal gyrus and cerebellum even after adjusting for age-related differences in accuracy, education and grey matter volume. Researchers have postulated that age-related increases in neural activation may be compensatory or due to neural dedifferentiation; where the increase must be related to performance to be considered compensatory [34, 35]. That some age-related increases in neural activation outside of the task network remained after adjustment for both accuracy and education supports neural differentiation [4].

This study extends lifespan findings of age-related decreases in task-related neural activation in episodic memory task areas [9, 12] and age-related increases in neural activation in areas deactivated by episodic memory [9, 11] to visuospatial working memory tasks. Previous studies comparing older and younger adults have also shown a similar pattern across numerous task paradigms (see [9]).

### Role of education

This study showed that lower education accounts for much of the relationship between age and decreased neural activation in the task associated network; age was associated with decreased activation in the task network, but this was not significant after adjustment for education. Interestingly, these results were significant even after controlling for grey matter volume.

The precuneus and middle frontal gyrus have been shown to be robust activations for spatial working memory tasks [36]. Previous studies have shown mixed results with respect to age-related differences in task-related neural recruitment; reporting increased activation with age, no difference between age groups and decreased activation with age [4], These discrepancies have been accounted for by the Compensation-Related Utilization of Neural Circuits Hypothesis (CRUNCH) [37]. This hypothesis suggests that increased neural recruitment is required for greater performance, and in older adults increased neural recruitment is required to reach equal performance to younger adults. However, when the task is too difficult and the older adults cannot perform to the same standard as the younger adults decreased neural activation can be observed [38–40]. Thus, for this task, decreased neural activation with age is consistent with the age-related decrease in task performance. That the decreases in neural activation were no longer significant after adjusting for education may indicate that education confers a greater ability to activate the neural network and subsequently improves task performance, suggesting that education effectively lowers the load of the task to the extent that the age-related difference in neural activation are no longer apparent.

Conversely, this study showed cubic age-related increases in the temporal gyrus, cuneus and cerebellum lobule VI, which all remained after adjustment for education. The temporal gyrus and cerebellum lobule VI have been implicated in visuospatial working memory, but are not reported as consistently as the precuneus and middle frontal gyri [36, 41–44]. Given the sigmoid shape to the cubic association this suggests that in the level of activation in this area is consistently low in the younger age group, increases with age for the middle age adults and plateaus in the older adults. Interestingly, a sigmoid relationship between neural activation and load has also been reported [39], demonstrating another parallel between increasing load and age-related neural changes.

Cognitive reserve describes adaptation to neural damage without cognitive symptoms [45]. Stern (2006) postulates cognitive reserve can occur in the brain in two forms: neural reserve and neural compensation. Neural reserve refers to brain networks less susceptible to structural effects of ageing, e.g. higher education and physical exercise have been associated with decreased age-related neural atrophy [46]. Neural compensation is where brain structures or networks functionally adapt to compensate for brain damage [45]. Higher education has been associated with greater grey matter volume and functional metabolism, which in turn was associated with better cognitive performance in healthy older adults [13] supportive of both the neural reserve and neural compensation models. Given that our study adjusted for grey matter volume throughout, these results are supportive of education providing a neural compensation component of cognitive reserve; education is associated with greater neural recruitment, thus greater task activation and better task performance even in the face of neural damage.

Taken together, these findings suggest that there is an increased neural recruitment in some less commonly task activated areas, mostly during the middle age range. In addition, there is a gradual age-related decrease in activation in areas more robustly activated during the working memory task, which is in turn associated with lower task performance. However, this decreased activation and subsequent poorer task performance is attenuated in those with higher education, consistent with both the CRUNCH [4, 37] and cognitive reserve models [33]. That the performance for the SAT correlated with many domains of two frequently employed neuropsychological tests demonstrates the potential for these results to be generalisable beyond just visuospatial working memory.

This indicates that in previous cross-sectional studies which have not controlled or matched for education the observed age-related decreases in task activation areas are likely to be partly age-related and partly due to lower education level and has implications for future neuroimaging ageing studies and the role of education in healthy ageing.

Future studies could investigate whether the increased activation in less commonly recruited task areas is related to maintaining performance in middle aged adults. Furthermore, as the increased activation plateaus in later life, it is possible that the continued age-related decrease in neural recruitment in more robustly task activated areas results in lowered performance in older age, despite compensatory recruitment, especially in those with lower education.

### Limitations

This study employed an fMRI task to address the role of education in working memory performance over the adult lifespan. However, there are some limitations. First, the ordinal measure of education may not provide as much sensitivity as years of education, similarly it lacks measures of quality of education which has been reported as important in cognitive reserve [47]. Second, this is a cross-sectional study and the relationship between age and education is a cohort effect. However, many ageing studies are cross-sectional and the conclusions demonstrating the impact of education on task performance and processing are still relevant to the interpretation of cross-sectional studies and future study design. Third, fMRI measures blood oxygen levels to indicate neural activity and can be considered a vascular rather than neural measure. Whilst the neural and vascular responses are known to be related in similar ways in young and older adults [48] blood circulation is likely to be affected by age and fitness, thus some reported age differences in fMRI studies may be related to differences in the haemodynamic response (HDR) rather than neural processing. Accordingly, past studies have shown greater variability in the HDR in older compared to younger participants [49, 50]. This study employed the internal control of a block design and using high and low demand task contrasts so that any differences can be more reliably interpreted as differences in cognitive processing as opposed to haemodynamic differences. Nonetheless, it is important to bear in mind the limits of fMRI interpretation. Fourth, whilst the study employed an innovative and sensitive method of adjusting for grey matter volume, there is evidence to suggest there are limitations to voxel-to-voxel adjustment as it does not account for the impact of lost grey matter in other areas of a relevant network, though it remains one of the more sensitive methods of adjustment to date [34, 51]. Finally, whilst the analysis of a full adult lifespan dataset is one of the main strengths of the study, the analysis is restricted to linear, quadratic and cubic models, which can be affected by the age range of the sample [52]. Previously we tested non-parametric smoothing splines on this dataset, but the non-parametric models tended to over fit the data, such that even linear associations were deemed insignificant. It was concluded that despite a worthy sample size there is insufficient data for non-parametric models to be informative at this stage.

### Conclusions

This study reported that increasing age was associated with linear decreases in task performance and activation. This relationship was confounded by education which was also associated with better performance. There were also non-linear increases in neural activation outside of the task positive network which were not associated with performance. The former findings suggest that education plays a primary role in increasing neural efficiency and thus improving task performance. The latter findings indicate that age is also associated with a non-linear increase in neural dedifferentiation which in some smaller non-task related areas can be attenuated by education. Taken together these findings suggest that education may play a minor active role in neural compensation.

In summary, these data support both linear and non-linear associations between age and neural function during spatial working memory performance, mirroring past studies employing episodic memory tasks. In addition, this study employed a sensitive adjustment for grey matter volume differences due to the effect of ageing on grey matter volume. Finally, this study demonstrated a role for education in cognitive reserve and highlights the importance of including education as a covariate in ageing studies.

## Supporting information

S1 Fig Neuropsychological scores by age. (DOCX)

S1 Table Detailed correlation matrix between age, education, neuropsychological measures and SAT performance. (XLSX)

S2 Table Detailed correlation matrix between age, neuropsychological measures and SAT performance adjusting for education. (XLSX)

S3 Table Detailed task activations. (XLSX)

S4 Table Detailed linear associations between task activations and age. (XLSX)

S5 Table Detailed cubic associations between task activations and age. (XLSX)
