# Supplementary material for: Working memory, age and education: A lifespan fMRI study
Source: PLoS One. 2018 Mar 27;13(3):e0194878. doi: 10.1371/journal.pone.0194878 (PMC5870997; doi:10.1371/journal.pone.0194878)
Supplement: S1 Fig — (DOCX) [file pone.0194878.s001.docx]

S1 Fig. Neuropsychological scores by age


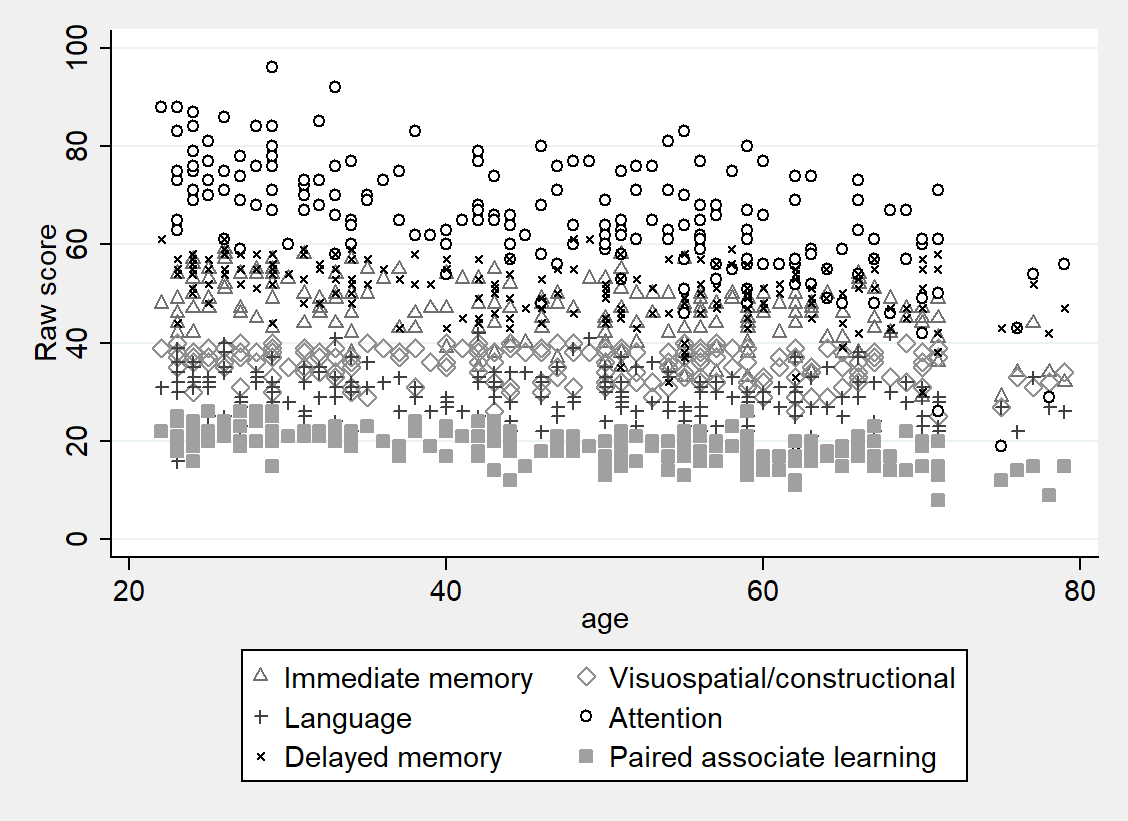


Raw scores (maximum score) for immediate memory (64), language (50), delayed memory (62), visuospatial/constructional (40), attention (105) as assessed by RBANS and paired associate learning first trial memory score (26) as assessed by CANTAB are displayed.
